# Effect of Flaxseed (*Linum usitatissimum* L.) Supplementation on Vascular Endothelial Cell Morphology and Function in Patients with Dyslipidaemia—A Preliminary Observation

**DOI:** 10.3390/nu14142879

**Published:** 2022-07-13

**Authors:** Dominika Kanikowska, Agnieszka Malińska, Agnieszka Mickiewicz, Agnieszka Zawada, Rafał Rutkowski, Krzysztof Pawlaczyk, Maki Sato, Andrzej Bręborowicz, Janusz Witowski, Katarzyna Korybalska

**Affiliations:** 1 Department of Pathophysiology, Poznan University of Medical Sciences, 60-806 Poznan, Poland; rrutkowski@ump.edu.pl (R.R.); abreb@ump.edu.pl (A.B.); jwitow@ump.edu.pl (J.W.); koryb@ump.edu.pl (K.K.); 2 Department of Histology and Embryology, Poznan University of Medical Sciences, 60-781 Poznan, Poland; amalinsk@ump.edu.pl; 3 1st Department of Cardiology, Medical University of Gdansk, 80-210 Gdansk, Poland; agnieszka.mickiewicz@gumed.edu.pl; 4 Department of Gastroenterology, Dietetics and Internal Diseases, Poznan University of Medical Sciences, 60-355 Poznan, Poland; a.zawada@ump.edu.pl; 5 Department of Internal Medicine, Nephrology and Transplantology, Poznan University of Medical Sciences, 60-355 Poznan, Poland; kpawlac@ump.edu.pl; 6 Institutional Research, School of Medicine, Aichi Medical University, Nagakute 480-1195, Aichi, Japan; msato@aichi-med-u.ac.jp

**Keywords:** flaxseed, endothelial cell, dyslipidaemia

## Abstract

Context: Flaxseed has a characteristic fatty acids composition and unique phytonutrient profile that may have health-promoting properties. Objective: This study aimed to determine the effects of 10 weeks of supplementation with the flaxseed (28 g/day) on endothelial cells (EC) function, serum lipids and proinflammatory mediators in patients with mild and severe dyslipidaemia. Materials and methods: Eleven lean patients with severe dyslipidaemia treated with apheresis (group 1; 10 weeks treated in four phases: (i) ordinary diet, (ii) ordinary diet + flaxseed, (iii) ordinary diet (wash out), (iv) ordinary diet + placebo) and eleven obese patients with mild dyslipidaemia—not treated with apheresis (group 2; 10 weeks treated in two phases: (i) ordinary diet, (ii) low fat diet + flaxseed). Flaxseed was given blindly. Serum was collected at the end of each phase of the study. ECs were exposed in vitro to the medium supplemented with pooled serum taken from patients from both groups to detect their morphological changes using light and electron microscopy. ECs proliferation was also measured at the end of each study phase. Results: Serum vascular endothelial growth factor was decreased after flaxseed supplementation but only in group 1. ECs proliferation was increased after flaxseed supplementation only in obese patients. ECs exposed to medium supplemented with obese patients’ serum revealed the following cellular abnormalities: accumulation of lipid droplets, changes of rough endoplasmic reticulum and mitochondria, and flaxseed did not reverse observed changes. At the same time, flaxseed supplementation decreases total cholesterol in both tested groups, low-density lipoprotein cholesterol in group 1 and triglycerides in group 2. Conclusions: Our findings support the potential role of flaxseed in treating dyslipidaemia but indicate only a slight impact on endothelial cell function.

## 1. Introduction

Flaxseed (*Linum usitatissimum* L.) appears to be a valuable source of polyunsaturated fatty acids (PUFAs), phytosterols and fibre, which lower lipids and have anti-atherogenic activity. Of PUFAs, flax comprises α-linolenic acid (ALA) and linoleic acid [1]. In particular, ALA and its metabolites are thought to exert beneficial cardiovascular effects [2,3]. Moreover, increased dietary α-linolenic acid (through flaxseed consumption) has been associated with decreased primary cardiovascular events, as well as a reduction in serum low-density lipoprotein cholesterol (LDL), elevated levels of which are a cardiovascular risk factor [4]—although results from human trials are inconsistent [5]. It is well established that flaxseed modified lipid concentration [6]. As we previously documented, flaxseed is also effective in lipid-lowering in patients with severe hyperlipidaemia undergoing lipoprotein apheresis. 

Flaxseed reduces total cholesterol, LDL, but not lipoprotein(a) Lp(a) [7]. Endothelial cell dysfunction and proinflammatory processes are closely related to the development of atherosclerosis and are risk factors for coronary artery disease (CAD) [8]. Processes like these are commonly found in those who have dyslipidaemia. The endothelium shows a significant role in regulating the degree of vascular tone, platelet adhesion, and aggregation, keeping the balance between coagulation and fibrinolysis, and others (for more details, see review [9]). Abnormalities of haemostasis and inflammation are associated with advanced atherosclerosis and more frequent cardiovascular events [10]. The early detectable atherosclerosis changes start with endothelial dysfunction, favouring the transfer and modification of circulating lipoproteins into the sub-endothelial space [11]. 

Askapour et al., 2020 performed a systemic review and meta-analysis on flaxseed supplementation in patients with obesity on proinflammatory cytokines and endothelial-specific adhesive molecules. The meta-analysis included a heterogeneous group of patients with various BMI and comorbidities, including CAD, diabetes mellitus (DM), metabolic syndrome, renal failure and others. It showed equivocal findings in inflammatory mediators and endothelial adhesive molecules [12]. The meta-analysis (40 randomised controlled trials; 2520 patients) revealed the effectiveness of flaxseed supplementation in reducing C-reactive protein (CRP), interleukin-6 (Il-6), and vascular cell adhesion molecule 1 (VCAM-1) and no effect on tumour necrosis factor-alpha (TNF-alpha) and E-selectin concentrations. The estimation of intercellular adhesion molecule 1 (ICAM-1) concentration showed significant study heterogeneity with the observation that flaxseed supplementation has no significant impact on serum ICAM-1 level [12].

Therefore, it is important to establish if flaxseed, a rich source of polyunsaturated fats, could be a valuable dietary option for individuals affected with dyslipidaemia. In addition, such a diet might have benefits for vascular endothelial function. With this respect, we intend to estimate endothelial cell function after flaxseed supplementation in patients with severe hyperlipidaemia treated with lipoprotein apheresis and obese patients with BMI > 30 kg/m^2^ and mild hyperlipidaemia.

Our experiments include detecting typical endothelial cell markers (VEGF, sICAM-1, sVCAM-1) in patients’ serum and in-depth morphological analysis of vascular endothelial cells, performed in light and electron microscopy. The morphological analyses were served after endothelial cell exposition to a medium supplemented with serum taken from patients treated/not treated with flaxseed in vitro conditions.

## 2. Methods

### 2.1. Study Design

**Figure 1:**
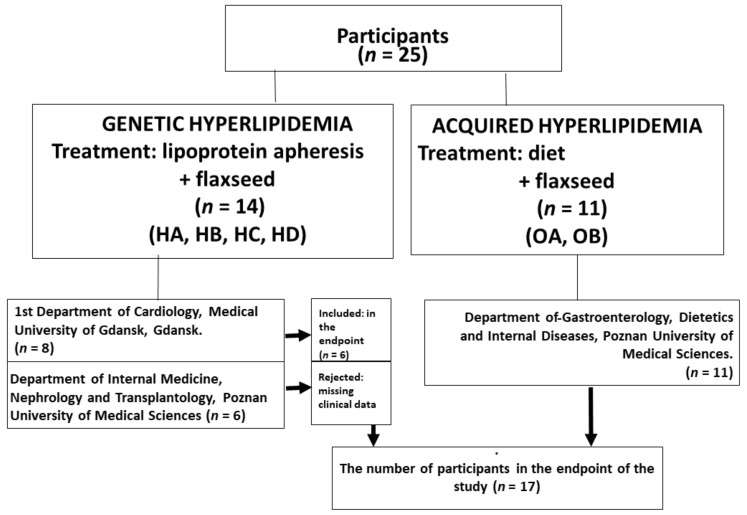
Diagram of the study. Abbreviation: HA—ordinary diet; HB—ordinary diet + flaxseed; HC—ordinary diet (wash out); HD—ordinary diet + placebo; OA—ordinary diet; OB—low fat diet + flaxseed.

**Figure 2:**
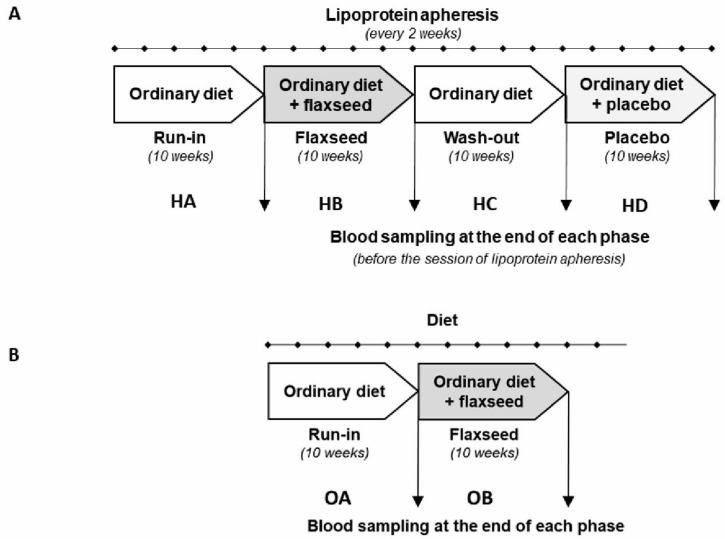
Study protocol (**A**)—Group 1 (HA, HB, HC, HD)—lipoprotein apheresis + flaxseed biscuits; (**B**)—Group2 (OA, OB)—treated with diet supplemented with flaxseed biscuits Abbreviation: H = hypercholesterolemia, O = obesity, A, B, C, D—the end of each phase.

This was a prospective study of two groups of patients with the following characteristics (Figure 1). The first group (*n* = 14) was composed of lean (*n* = 6), and obese (*n* = 8) outpatients receiving therapy of lipoprotein apheresis (once every two weeks) for severe hyperlipidaemia were registered with a single arm and the phases of treatment and placebo in a pre-determined single sequence. These participants undertook an initial 10-week run-in phase on a standard diet (HA), after which they obtained flaxseed supplementation for 10 weeks (HB). This was followed by a washout period of 10 weeks on a standard diet (HC). Afterwards, patients received a placebo supplementation for 10 weeks (HD) (Figure 2A). The participants were blinded as to what type of supplementation they received. The second group was composed of 11 obese outpatients with dyslipidaemia receiving a diet at home (OA) with a single arm and the phases of treatment (OB) (Figure 2B). The participants consumed a low-fat and low carbohydrate diet (45% carbohydrates, 15% proteins, 40% fat). Lifestyle was not controlled during the treatment. This group was not treated with apheresis. In both groups, flaxseed was given blindly.

The primary endpoint was the abnormalities in (1) endothelial cell functions assessing serum concentration of VEGF, sICAM-1, sVCAM-1, (2) endothelial cell proliferation, and (3) morphology after cells exposition to medium supplemented with serum taken from patients at the end of each stage of the experiment.

Secondary endpoints were: (1) The concentration of inflammatory mediators: high-sensitivity IL6 (hsIL-6), high-sensitivity interleukin 1 beta (hsIL-1beta), high-sensitivity CRP (hsCRP) and high-sensitivity TNF alpha (hsTNF alpha); (2) the concentrations of total cholesterol (TC), oxidised low density lipoprotein cholesterol (oxy-LDL), high-density lipoprotein cholesterol (HDL-C), low density lipoprotein cholesterol (LDL-C), triglycerides (TG); and (3) tolerability.

### 2.2. Study Population

A total of 25 patients receiving diet or lipoprotein apheresis (once every two weeks) for severe hyperlipidaemia were registered. Written informed consent was provided for all patients before they entered the study. Recommendations for lipoprotein apheresis (group 1) was precisely described elsewhere [7].

All patients treated with lipoprotein apheresis were from the Department of Cardiology of Medical University of Gdansk and the Department of Nephrology of Poznan University of Medical Sciences, one of the two leading centres in this respect. However, three patients discontinued treatment and did not complete the survey because of and lack of genetic test results of disorders diagnosis (*n* = 3). For the planned endpoints, the data from 22 per-protocol patients were studied. The inclusion criteria for obese patients (group 2) involved age above 18 years, BMI ≥ 30 kg/m^2^, total cholesterol >150 mg/dL but without lipid-lowering medicines. The exclusion criteria involved overt malignant or systemic illness, pregnancy, acute coronary syndrome over the past six months, congestive heart failure, bariatric surgery, a known eating disorder, and a change in body weight greater than 2 kg over the past three months.

The research was approved by the Ethics Committee of the Poznan University of Medical Sciences (No. 333/15) and followed the principles of the Declaration of Helsinki. Patients’ baseline characteristics are provided in Table 1.

### 2.3. Flaxseed Supplementation and the Study Protocol

Flaxseed was given for 10 weeks at a dose of 28 g/d, according to the study by Dittrich et al. [13] and Pan et al. [14]. Ground flaxseed was administered in biscuits, based on the idea of Wong et al. [15]. Cookies were created and baked especially for the project, and the experimental biscuits’ composition was precisely described elsewhere [7]. Biscuits used as a placebo contained the same amount of whole wheat flour instead of flaxseed. Patients were supervised by a qualified dietician and consumed the required number of biscuits at the same time of day (mid-morning).

For participants in group 1 (Figure 2A), all nutritional interventions were carried out in addition to regular lipoprotein apheresis treatment and lipid-lowering medications, which did not change during the study period. 

Patients from group 2 (Figure 2B) carried regular medications and diet without lipid-lowering medicines. Patients had blood sampled on 4 or 2 occasions (Figure 2A,B). The university hospital laboratory performed routine biochemical tests.

### 2.4. Study Limitation

Only a few centres in Poland provide lipoprotein apheresis for severe hyperlipidaemia, and each centre treats only a few patients. As a result, the study suffered from a small sample size due to the patients’ recruitment. Although our analysis was highly rigorous in design, the patients’ daily diet, sleep patterns and physical activity were not controlled. All of these differences in lifestyle could affect results.

### 2.5. Biochemical Analyses

Fasting blood samples were always collected between 07:30 and 09:00 am to minimise diurnal variations. All routine biochemical analyses were performed immediately in a central hospital laboratory. Serum lipids and glucose were analysed according to the standard laboratory techniques. Serum samples for endothelium and biochemical measurements were aliquoted and stored at −80 °C until assayed in batch. Concentrations of hsIL-6, adiponectin, leptin and hsCRP were determined by immunoassays (BioVendor, Brno, Czechia), concentrations of hsTNF alpha, VEGF, sICAM-1, sVCAM-1 and hsIL-1 beta were measured by immunoassays (R&D Systems, Minneapolis, MN, USA) and oxy-LDL was determined by immunoassays (Immunodiagnostik AG, Bensheim, Germany). All tests were performed as per manufacturers’ instructions. 

The sensitivity of the assays was: 0.32 pg/mL for hs IL-6, 0.47 ng/mL for adiponectin, 0.2 ng/mL for leptin, 0.02 µg/mL for hsCRP, 0.049 pg/mL for hsTNFα, 12.2 pg/mL for VEGF, 17.5 pg/mL for sICAM-1, 10.7 pg/mL for sVCAM-1, 0.033 pg/mL for hsIL-1 beta, 4.13 ng/mL for oxy-LDL.

### 2.6. Cell Culture

Human umbilical vein endothelial cell (HUVECs) line EA.hy926 (kindly provided by Dr. CJ Edgell, University of North Carolina, Chapel Hill, NC, USA) was used in in vitro experiments [16]. The cells were cultured in the Earl’s-buffered M199 culture medium, supplemented with gentamycin (50 μg/mL), amphotericin (2.5 μg/mL), L-glutamine (2 mmol/L), hydrocortisone (0.4 μg/mL) and 10% *v*/*v* foetal calf serum (Invitrogen, Waltham, MA, USA). The cells were incubated at 37 °C in a humidified atmosphere of 95 % air and 5% CO2. Cell culture plastics were bought from (Nunc, Roskilde, Denmark) and (Costar, Glendale, AZ, USA), whereas reagents were purchased from (Sigma-Aldrich, St. Louis, MO, USA).

### 2.7. Experimental Design

The experimental model was performed as described previously [17]. Endothelial cells were exposed for one and seven days to the culture medium supplemented with 10% *v*/*v* foetal calf serum (FCS) and 10% human pooled serum. Human pooled serum was collected from an identical volume of serum taken from 11 patients with severe dyslipidaemia (H) receiving lipoprotein apheresis (points: HA, HB, HC, HD—see flow chart Figure 2A), 11 obese patients (O) with mild hyperlipidaemia (points: OA and OB—see flow chart Figure 2B) and 10 healthy controls. Healthy control individuals (*n* = 10) were recruited from regular blood donors of the Regional Blood Donation Center in Poznań. They included 5 women in average age 45.2 ± 1.6 (mean ± SD) and BMI 23.8 ± 1.5 and 5 men in age 44.0 ± 1.8 and BMI 28.4 ± 1.8.

The culture medium was supplemented with the pooled human serum in 10% *v*/*v*. 

The formed medium was then sterilized with siring filters (0.22 µm pores, Millipore, Burlington, MA, USA). The sterilization process protects cultured endothelial cells against possible infection, especially during extended exposition (7 days). The medium was exchanged every three days. In our experiments, we used two types of controls. One control consists of a medium supplemented with 10% *v*/*v* foetal bovine serum. This kind of medium is recommended for HUVECs culture, as described in the method section (con = standard medium). The second control consists of a medium supplemented with 10% *v*/*v* human serum from healthy, lean patients of matched age and sex (HS). The second control is not a physiological milieu for the HUVECs and was prepared for the experiment to evaluate the heterogeneity of human serum.

### 2.8. Endothelial Cell Proliferation

HUVECs were exposed to a medium supplemented with individual human serum in triplicates (HA, HB, HC, HD—11 patients, OA. OB—11 patients) and standard culture control (con; *n* = 10) for 24 h to assess cell proliferation, using the MTT assay. In the MTT test, active mitochondrial dehydrogenases convert the MTT salt (3-[4,5-dimethylthiazol-2-yl]-2,5-diphenyl-tetrazolium bromide) to formazan product [18]. The test was performed as described previously [19]. Briefly, endothelial cells were seeded at 5000 cells/well density and exposed for 24 h to tested media. Then cells were incubated in a medium containing 1.25 mg/mL of the MTT salt for 4 h at 37 °C. The formazan product was dissolved using an acidic solution of 20% *w*/*v* sodium dodecyl sulphate and 50% *v*/*v* N, N-dimethylformamide. The optical density of obtained dye was recorded at 595 nm. The data were expressed as a percentage of control (standard medium).

### 2.9. Light Microscopy

HUVECs were seeded in 24 wells plates and incubated with standard medium (con) and medium supplemented with pooled human sera (HA, HB, HC, HD, OA, OB, and HS) for one and seven days. Every 1–3 days using the Axio Observer D1 inverted microscope (Zeiss, Oberkochen, Germany), the microphotographs were taken up to the seven days of the exposition.

### 2.10. Electron Microscopy

Endothelial cells were harvested using trypsin/EDTA after one and seven days of exposition to standard medium (con) and medium supplemented with pooled human sera (HA, HB, HC, HD, OA, OB and HS) and then processed according to method precisely described elsewhere [17]. 

### 2.11. Statistical Analysis

All statistical analyses were performed using GraphPad Prism 8.0 (GraphPad Software, La Jolla, CA, USA). The normality of the data distribution was tested with the D’Agostino Pearson test, and the statistical analysis was chosen according to Gaussian/non-Gaussian distribution. The results were interpreted with repeated measures analysis of variance using a post hoc test for multiple comparisons or a *t*-test for paired/unpaired data.

The results are presented as individual data with the medians and interquartile ranges. A value of *p* < 0.05 was considered significant.

## 3. Results

### 3.1. Patients Characteristics

All patients with severe dyslipidaemia (group 1): familial hypercholesterolemia (*n* = 4), statin intolerance (*n* = 2), isolated hyperlipoprotein (a) (*n* = 2) and mixed hyperlipidaemia (*n* = 5), require lipoprotein apheresis—the treatment of choice in patients with the severe hyperlipidaemia. The obese patients with mild dyslipidaemia (group 2) (*n* = 11) undergo lifestyle changes, including dietary modification to reduce saturated fats, trans-fats, cholesterol and carbohydrates. Obese patients with mild dyslipidaemia (group 2) have about 27% higher BMI (*p* = 0.0001) and lower LDL cholesterol concentration (*p* = 0.0157). There were no differences in other lipids parameters between these two study groups. Obese patients from group 2 also have 4.6 times higher leptin concentration than lipoprotein-apheresis treatment patients (group 1) (*p* = 0.0031). There were no significant differences in other measured parameters (Table 1).

### 3.2. Effect of Flaxseed on Endothelial Cells’ Biomarkers

VEGF concentration was lower (*p* = 0.0246) after treatment with flaxseed (HB) compared with the run-in phase (HA) by 27% (Table 2). This effect was seen only in the patients with severe dyslipidaemia treated with lipoprotein-apheresis (group 1). There was no significant effect of flaxseed supplementation on serum levels of ICAM-1 and VCAM-1 in both groups of patients (Table 2).

### 3.3. Effect of Flaxseed on Endothelial Cell Morphology

#### Light Microscopy 

**Figure 3:**
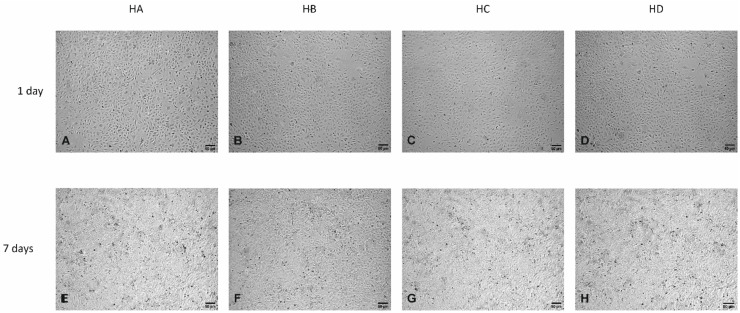
Light microphotographs of HUVEC line EA.hy926 after one (**A**–**D**) and seven days (**E**–**H**) of exposition to medium supplemented with: human pooled serum taken from a patient with genetically determined hypercholesterolaemia collected in: the regular diet phase (HA) after ten weeks of flaxseed substitution (HB), during the “wash out” phase (HC), after ten weeks of taking placebo (HD). Appropriate controls are presented on thy Figure 4 (**A**, **B**; one day, **E**, **F**; seven days). Magnification 100×.

**Figure 4:**
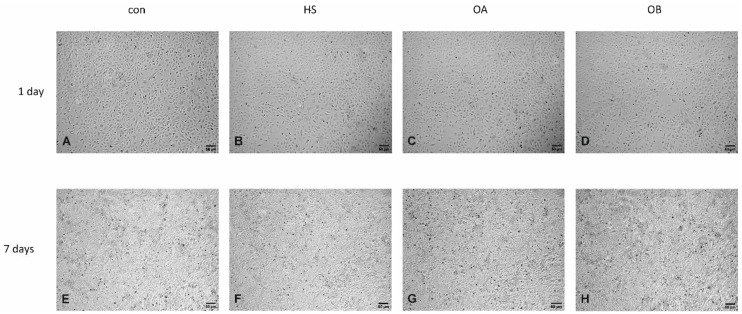
Light microphotographs of HUVEC line EA.hy926 after one (**A**–**D**) and seven days (**E**–**H**) of exposition to medium supplemented with: foetal calf serum (CON), human serum taken from healthy patients (HS), human pooled serum taken from obese patients with dyslipidaemia before the administration of flaxseed (OA), human serum taken from obese patients with dyslipidaemia after a 10-weeks dietary supplementation with flaxseed (OB). Magnification 100×.

No morphological changes were observed in HUVECs line EA.hy926 after one (Figure 3A–D; Figure 4A–D) and seven days of exposition (Figure 3E–H; Figure 4E–H) to medium supplemented with human serum taken from patients with severe dyslipidaemia (points A, B, C, D) and obese patients with mild dyslipidaemia (points A, B), treated/not treated with flaxseed, using light microscopy. In all tested groups, ECs maintained the typical cobblestone appearance (Figure 3A–H, Figure 4A–H). Tested cells exposed to medium supplemented with hypercholesteraemic sera and sera taken from obese patients exhibited the same cobblestone appearance compared to two tested controls (con = standard medium—Figure 4A,E; human serum taken from lean, healthy patients with matched age = HS—Figure 4B,F).

### 3.4. Electron Microscopy

**Figure 5:**
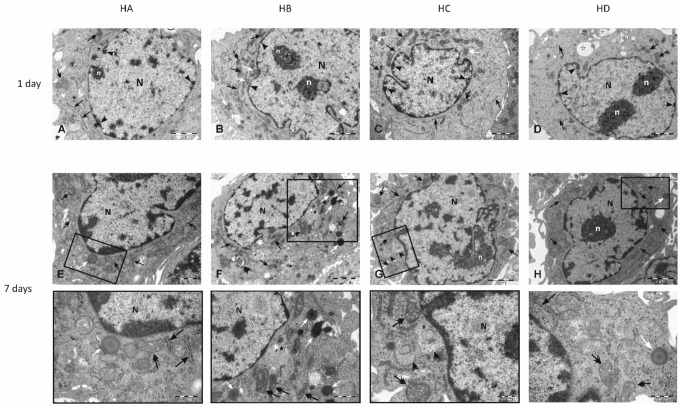
Electron micrographs of EA.hy926 cell line derived from human umbilical vein endothelial cell (HUVEC) after one (**A**–**D**) and seven days (**E**–**H**) of exposition to medium supplemented with: human pooled serum taken from a patient with genetically determined hypercholesterolaemia collected in the regular diet phase (HA), after ten weeks of flaxseed substitution (HB), during the “wash out” phase (HC), after ten weeks of taking placebo (HD). One day of exposure to a modified medium (**A**–**D**) did not cause any relevant differences in the cell ultrastructure between study groups. A pale cytoplasm contains numerous, small mitochondria with dark matrix and densely packed, intact cristea (double head arrows), typical architecture of rough endoplasmic reticulum (RER, black arrows) and single lipid droplets (white arrows). In the cytoplasmatic area of cells belonging to the HA group multivesicular bodies were present (black star), while in sections derived from cells taken from HD group vacuolated secretory granules were observed (white star). In all analysed groups, nucleus (N) is irregular in shape and contains a small portion of heterochromatin located at the periphery (arrowheads). n—nucleolus. Seven days of exposition to modified medium did not reflect the significant changes in the ultrastructural composition of HUVECs derived from HA, and HD groups (**E**,**H**). In cytoplasm an irregular in shape nucleus with heterochromatic rim, mitochondria (double head arrows) with typical ultrastructural features, not abundant RER (black arrows) and individual lipid droplets (white arrows) were observed. n- nucleolus. Insets present magnified region of cytoplasm with typical, unchanged ultrastructural components. Ultrastructural changes in HUVECs derived from the HB group (**F**) refer mainly to mitochondria which showed a condensed conformation with enlarged intra-cristal spaces (double head arrows). In the cytoplasm, poorly developed RER (black arrows), lipid droplets (white arrows) and individual autophagic vacuole (double arrowhead) can be observed. Note multivesicular bodies present in close proximity to the nucleus and the peripheral region of the cytoplasm (black stars). The inset presents a magnified area of cytoplasm with modified mitochondria and lipid droplets. Changes in mitochondrial architecture (double head arrows) were also observed in cells derived from the HC group (**G**). In the cytoplasm, individual RER cisterns (black arrows) and autophagic vacuoles with an amorphous sub-cellular material were present (double arrowhead). n—nucleolus. The inset presents the cytoplasm’s magnified region located in the nucleus’s close vicinity, where modified mitochondria and vacuoles were observed. Appropriate controls are presented in Figure 6 (**A**,**B**; one day, **E**,**F**; seven days).

**Figure 6:**
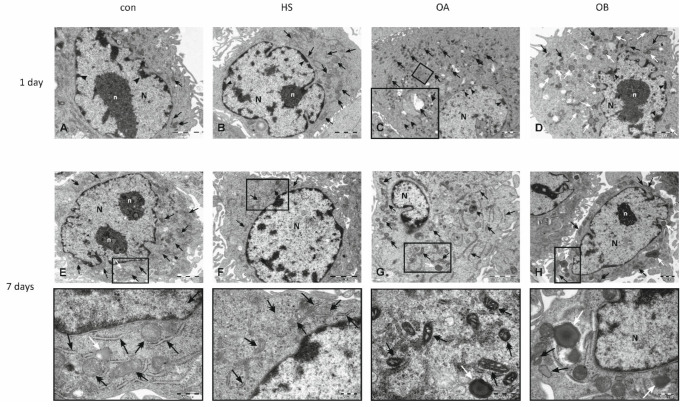
Electron micrographs of EA.hy926 cell line derived from human umbilical vein endothelial cell (HUVEC) after one (**A**–**D**) and seven days (**E**–**G**) of exposition to medium supplemented with: foetal calf serum (CON), human serum taken from healthy patients (HS), human pooled serum taken from obese patients with dyslipidaemia before the administration of flaxseed (OA), human serum taken from obese patients with dyslipidaemia after a 10 weeks dietary supplementation with flaxseed (OB).The ultrastructural composition of HUVECs derived from CON and HS groups did not show any spectacular changes after both one (**A**,**B**) and seven days (**E**,**F**) of culturing. Organelles are dispersed in the cytoplasm and represent a physiological cellular state. Nucleus (N) with a characteristic homochromatic rim is localised in the central area of the cytoplasm. Mitochondria with electron-dense matrix and densely packed cristae (double head arrows), cistern of rough endoplasmic reticulum (RER, black arrows) and individual lipid droplets (white arrow) were observed. n—nucleolus. Insets present an enlarged cytoplasm region showing the normal ultrastructural features of mitochondria, RER, and lipid droplet presence. In HUVECs derived from the OA group, ultrastructural alterations were observed in both time intervals. After one day of exposure to a modified medium (**C**) in the cytoplasm, numerous mitochondria with normal ultrastructural architecture were present (double head arrows). In the cytoplasm’s central area, an astonishing amount of RER cisterns (black arrows) were observed. Arrowheads—peripheral accumulation of heterochromatin in the nucleus (N). Inset shows high magnification of RER where features of the degranulation process are demonstrated. The changes in the ultrastructure of cells after seven-day exposure to the modified medium (**G**) concerned the structure of mitochondria - small, with an electron-dense matrix and enlarged intra-cristal spaces (double head arrows) primarily. The other organelles represented their typical physiological features: nucleus with the well demonstrated peripheral presence of heterochromatin (n), normal organisation of RER (black arrows), individual lipid droplet (white arrow). Inset shows the enlarged area of cytoplasm where enlarged intra-cristal spaces in mitochondria are visible) and lipid droplet is also present. Electron microphotograph of HUVECs derived from OB group after one day of culture in modified medium (**D**) shows singular mitochondria (double head arrows) dispersed in the whole area of cytoplasm, poorly developed RER (black arrows) and numerous lipid droplets (white arrow) present in the whole area of cytoplasm. Nucleus (N) with well-developed nucleolus (n) and heterochromatic rim (arrow heads) demonstrated irregular shape. Ultrastructural changes in HUVECs from OB group after seven days of exposure to the modified medium (**H**) were reflected mainly in a large number of lipid droplets (white arrows) bigger than structures observed in cells after one day of culture. Small mitochondria (double head arrows) and singular RER cisterns (black arrows) are dispersed in the cytoplasm’s peripheral region. N—nucleus, n—nucleolus. The inset demonstrates a magnified part of cytoplasm with lipid droplets, the normal mitochondria and RER structure.

Transmission electron microscope studies were performed to determine ultrastructural changes in HUVECs morphology. After one and seven days of exposure to the modified medium, no significant differences between study groups in the cell ultrastructure were observed in patients with severe dyslipidaemia (group 1) (HA—Figure 5A,E; HB—Figure 5B,F; HC—Figure 5C,G; HD—Figure 5D,H) and controls (con—Figure 6A,E; HS—Figure 6B,F). Whereas ECs exposed to medium supplemented with serum taken from obese patients (group 2) revealed changes in cellular ultrastructure in both time intervals (OA—Figure 6C,G; OB—Figure 6D,H). The ultrastructural changes were generally concerned with the (i) accumulation of lipid droplets in the cytoplasm, (ii) changes of mitochondrial architecture reflected by enlarged intra-cristal spaces and (iii) decreasing amount of rough endoplasmic reticulum (RER) cisterns. In the cytoplasm, autophagic vacuoles were also observed. These changes reflect endothelial metabolic and energetic dysfunction, and ten weeks with flaxseed supplementation did not reverse them. All observed details in electron micrographs are precisely described in Figure 5 and Figure 6.

### 3.5. Effect of Flaxseed on Endothelial Cell Proliferation

**Figure 7:**
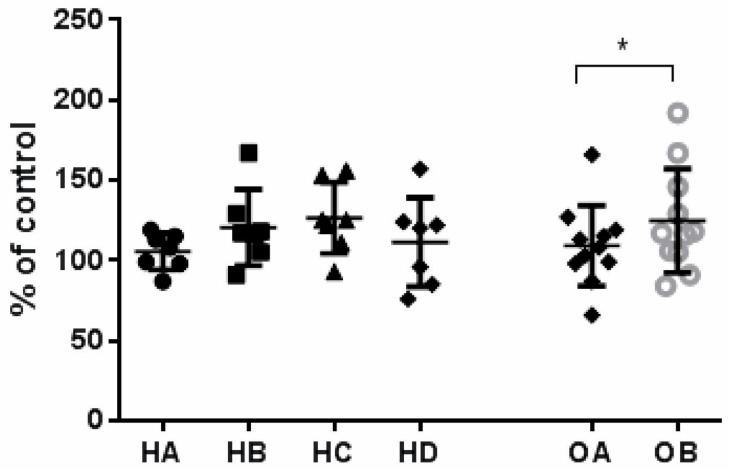
Endothelial cell proliferation after 24h exposition to medium supplemented with serum taken from individuals with genetically determined severe hyperlipidaemia (*n* = 6) collected in the regular diet phase (HA), after ten weeks of flaxseed substitution (HB), collected during the “wash out” phase (HC), collected after ten weeks of taking placebo (HD), taken from obese patients (*n* = 11) before the administration of flaxseed biscuits (OA), taken from obese patients after a 10 weeks dietary supplementation with flaxseed (OB). The data were expressed as percent of standard control (con = cells maintained in cultured medium supplemented with foetal calf serum) and derived from two independent experiments. Data were analysed using the *t*-test for the matching pairs (OA, OB). HA, HB, HC and HD were statistically analysed using one-way ANOVA. Statistical significance: * *p* < 0.05. OA vs. OB.

Endothelial cell proliferation after exposition to medium supplemented in human serum was slightly (+14.1%) but significantly increased only in obese patients with mild dyslipidaemia after diet supplemented with flaxseed biscuits (OB) compared with run-in-phase (OA) (Figure 7).

We did not observe any changes in endothelial cell proliferation in patients treated with lipoprotein apheresis supplemented with flaxseed biscuits (Figure 7).

### 3.6. Effect of Flaxseed on Parameters of Inflammation

There was no significant effect of flaxseed supplementation on hsCRP, hsTNFα, hsIL-1beta and hsIL-6 levels in both groups of patients (Table 3). The results of group 1 patients were previously shown in Table 2 *Nutrients*, 2020 [7].

### 3.7. Effect of Flaxseed on TC, LDL, Oxy-LDL, HDL and TG Levels

Total cholesterol and triglycerides levels were significantly lower after treatment with flaxseed (OB) compared with the run-in phase (OA) (Table 3). The median cholesterol level was lower by 9.2% (*p* = 0.0225), and the median triglycerides level was lower by 25.1% (*p* = 0.0137). In contrast, there was no significant difference between the treatments in oxy-LDL, HDL levels (Table 2 and Table 3). A similar effect was also seen in patients with severe hyperlipidaemia (group 1) (reduced LDL and TC concentration). The results were previously shown in *Nutrients*, 2020 [7].

### 3.8. Side Effects and Tolerability

Flaxseed and placebo cookies were well-tolerated and no other adverse effects were reported.

## 4. Discussion

Patients diagnosed with dyslipidaemia who took 28.0 g daily of flaxseed showed a significant reduction in serum lipids but exhibited a slight beneficial effect on endothelial cells. These effects might be partly explained by the lack of flaxseed influence on proinflammatory mediators in severe dyslipidaemia patients [7] and obese patients with mild dyslipidaemia, moreover with no impact on oxy-LDL, a crucial factor responsible for endothelial dysfunction [20]. The early detectable changes in the endothelium under the influence of atherosclerosis were caused by the transfer and modification of circulating lipoproteins to the sub-endothelial space. The inflammatory process accompanying atherosclerosis activates the endothelium. It changes ECs phenotype towards vasoconstriction, pro-inflammation and pro-thrombosis. The activation of the cell surface receptors could drive atherogenic endothelial phenotype by circulating inflammatory mediators and oxy-LDL [21]. Hypercholesterolemia and a high concentration of oxy-LDL can reduce the level of endothelium-derived nitric oxide (NO). NO is responsible for endothelium-dependent relaxation and plays a central role in the atherosclerotic process [9].

Flaxseed supplementation in patients with severe dyslipidaemia treated with apheresis (group 1) caused a decrease in VEGF concentration. VEGF is a potent stimulator of angiogenesis, which is crucial in cancer progression. Bergman et al. study suggested that flaxseed and its lignans have powerful antiestrogenic effects on oestrogen receptor-positive breast cancer in mice and may decrease VEGF in vivo and in vitro experimental design studies [22]. When analysing both study groups, we did not find changes in sICAM-1 and sVCAM-1 level and other endothelial proinflammatory markers. These observations are consistent with data presented in our previous paper showing no flaxseed effect on proinflammatory markers (Il-6, CRP, TNF-α) [7]. A meta-analysis by Askapour et al. reached partially similar conclusions and determined that flaxseed supplementation has no significant impact on serum ICAM-1, TNF—α and E-selectin. They also reported flaxseed’s impact on reducing VCAM-1, CRP and Il-6 levels. Such a variety of results could be explained by meta-analysis study-heterogeneity: (i) Comorbidities including patients with diabetes, CVD, metabolic syndrome, hypercholesterolemia, etc.; (ii) varied patients’ BMI ranging from 25.1 to 47.1; (iii) duration of flaxseeds treatment from 3 to 54 weeks [12].

In vitro experiments revealed no effects of flaxseed diet on endothelial cell proliferation after cell exposition to medium supplemented with sera taken at the end of each phase of treatment from severe hyperlipidaemia patients (group 1). Additionally, light and electron microscopy observed no ultrastructural changes in endothelial morphology. This effect observed only in patients with genetically determined severe dyslipidaemia may be partially explained by their serum seemed to be a more gentle milieu (lean patients treated every two weeks with apheresis) for in vitro EC growth when compared with serum taken from obese patients filled with proinflammatory adipokines with documented adverse effects on the endothelium [20,23]. Slightly different results were observed in obese patients with mild dyslipidaemia (group 2). Alterations in endothelial cells detected in vitro culture were more pronounced than those with genetically determined severe dyslipidaemia. These ECs’ structural modifications might be explained because obesity is the primary factor of endothelial dysfunction [20].

Moreover, we observed a slight increase in endothelial proliferation in those patients after flaxseed treatment. After exposition to medium supplemented in human serum, endothelial cell proliferation was significantly increased only in obese patients with mild dyslipidaemia. There are only a few reports on the relationship between endothelium proliferation and the effectiveness of a diet. Korybalska et al. focused their studies on the efficacy of a low-calorie diet, considering the subjects’ BMI. She found that endothelial proliferation increased with body weight loss [24]. Moreover, Korybalska et al. reported a significant decrease in endothelial cell proliferation, which correlates with the magnitude of weight loss only in men, not in women. This effect corresponded with serum levels of leptin and adiponectin but was not related to serum concentrations of several known proangiogenic mediators such as VEGF, MCP-1, TSP-1, MMP-9 and angiopoietin-2 [25]. Our study addressed this issue in a diet with flaxseed supplementation but without changes in body weight. Thus, flaxseed supplementation may be more effective in obese patients than those with severe hyperlipidaemia in improving ECs function.

Furthermore, we observed several ultrastructural changes in cell morphology which were only seen in electron microscopy but not in light microscopy. These changes were generally concerned with the accumulation of lipid droplets in the cytoplasm, changes in mitochondrial architecture and rough endoplasmic reticulum (RER) cisterns. These changes can reflect endothelial metabolic and energetic dysfunction, and flaxseed supplementation did not reverse them. Lipids are transported to the organelles and cellular membranes via vesicles of the secretory pathway. In the past decade, several studies demonstrated that metabolic products such as glucose and lipid oxidation products, including oxysterols, could contribute to endoplasmic reticulum stress with subsequent progression of atherosclerosis [26]. Mitochondria generate most of the cell’s energy (ATP) during oxidative phosphorylation and are also involved in cellular signalling, differentiation, proliferation, apoptosis and cell death [27].

Changes in mitochondrial architecture were visualised by electron microscopy studies, which allows us to analyse the temporal dynamics of mitochondrial function in living specimens [28]. It is well known that there is a close physical and functional association between mitochondria and the endoplasmic reticulum (ER) membranes. Dysfunctions in this interface between the mitochondria and the ER can lead to disturbances in cellular homeostasis, especially in coordinating biological functions, such as calcium ion signalling, regulation of apoptosis or ER stress response [29]. The presence of multivesicular bodies can reflect a desperate strategy for cell survival under stressful conditions caused by disturbance of cellular metabolic balance [30].

Finally, we observed reduced TC and LDL concentrations in severe dyslipidaemia patients due to (i) apheresis, (ii) flaxseed supplementation and lipid-lowering treatment. Like the group with severe dyslipidaemia, flaxseed supplementation in obese patients also decreased lipid concentration, especially TC and TG, without any lipid-lowering treatment support. Our data concerning serum lipids are consistent with a growing body of work proposing a protective effect of flaxseed against dyslipidaemia [7,31]. Vegetable oils are one of the essential components of the daily diet. They are a source of *n*-6 and *n*-3 PUFAs or monounsaturated fatty acids (MUFA), the consumption of which can improve endothelial function, have anti-inflammatory properties, and lower triglyceride concentrations [32,33].

Flaxseed is characterised by a high content of PUFA and is also the richest source of lignans in vegetables. Thus, the PUFA omega-3 family, dietary fibre and phytoestrogen lignans determine flaxseed’s hypolipidemic and anti-atherogenic actions.

Finally, gastrointestinal or other adverse effects were not reported during the study, suggesting that this dose of flaxseed is safe when administered for a few months.

## 5. Conclusions

At this stage, we cannot conclude that a diet containing flaxseed produces beneficial changes in the vascular endothelium of patients with dyslipidaemia. The present study shows only a tendency that flaxseed might have the potential ability to improve endothelial function but, in obese patients, have no potency to reverse endothelial ultrastructural changes observed in electron microscopy. The current project opens up new possibilities regarding the benefits of a diet containing flaxseed, emphasising the impact of such a diet on vascular endothelial function and Ecs morphology. The main observation of the present study is that flaxseed supplementation produced consistent lipid-lowering effects and can modulate VEGF concentration depending on the severity of dyslipidaemia. 

## Figures and Tables

**Table 1:** Baseline characteristics of the participants. The data are given as medians and range, *n* = 17. Abbreviations: BMI, body mass index; HDL, high density lipoprotein; LDL, low density lipoprotein; hsCRP, high-sensitivity C-reactive protein; hsIL-1β, high-sensitivity interleukin 1β; hsIL-6, high-sensitivity interleukin 6; hsTNFα, high-sensitivity tumour necrosis factor α; F, female; M, male; Y, yes; N, no; NA, not applicable. The data were analysed with Wilcoxon signed-rank test and a chi-square test.

	GROUP 1 (Treated with Apheresis + Flaxseed Biscuits) *n* = 11	GROUP 2 (Treated with Diet + Flaxseed Biscuits) *n* = 11	*p* Value
Sex, M/F	7/4	1/10	
Age, years	51.0 (29.0–79.0)	60.0 (35.0–73.0)	0.5612
BMI, kg/m^2^	25.6 (18.2–32.0)	35.5 (31.6–47.6)	0.0001
Underlying condition:			
1. Isolated hyper-Lp(a)	2	NA	
2. Familial hypercholesterolemia (confirmed)	2	NA	
3. Clinical phenotype of familial hypercholesterolemia	2	NA	
4. Statin intolerance	2	NA	
5. Mixed hyperlipidaemia	5	NA	
Dyslipidaemia, Y/N	11/0	11/0	
Coronary artery disease, Y/N	7/4	0/11	
Stroke, Y/N	2/9	0/11	
Diabetes, Y/N	1/10	3/8	
Hypertension, Y/N	5/6	8/3	
Time on Lipoprotein apheresis, months	26.5 (19.0–38.0)	NA	
Lipid profile and proinflammatory markers before commencement of lipoprotein apheresis/diets:			
Total cholesterol, mg/dL	239.5 (191.0–367.0)	239.0 (178.0–271.0)	0.0591
LDL, mg/dL	152.8 (118.0–152.5)	150.7 (102.4–182.4)	0.0157
HDL, mg/dL	49.0 (39.0–77.0)	52.0 (41.0–65.0)	0.0940
Triglycerides, mg/dL	294.5 (187.0–735.0)	167.0 (76.0–267.0)	0.4421
Leptin, ng/mL	15.0 (2.3–89.6)	71.4 (18.2–156.4)	0.0031
Adiponectin, ng/mL	438.5 (161.8–648.1)	598.1 (258.6–804.1)	0.1330
hsCRP, µg/mL	482.5 (301.0–2930.0)	400.4 (141.5–798.3)	0.8404
hsIL-1β, pg/mL	0.444 (0.240–0.992)	0.382 (0.339–0.669)	0.4479
hsIL-6, pg/mL	10.0 (7.7–37.0)	9.3 (7.6–25.4)	0.4299
hsTNFα, pg/mL	0.674 (0.392–2.68)	0.958 (0.517–1.53)	0.0754
Additional medication:			
1. Statins, Y/N	6/5	0/11	
2. Ezetimibe, Y/N	6/5	0/11	
3. Fenofibrate, Y/N	1/10	0/11	

**Table 2:** Changes in endothelial parameters and oxy LDL throughout the study. The data are given as medians and range. Abbreviations: VEGF, vascular endothelial growth factor; sICAM, soluble intercellular adhesion molecule-1; sVCAM, soluble vascular cell adhesion molecule-1, oxy-LDL, oxy-low density lipoprotein cholesterol. The data were analysed with Friedman test with Dunn post hoc test. Wilcoxon signed-rank test.

Parameters	Group 1 (*n* = 11)					Group 2 (*n* = 11)		
	HA (Run in)	HB (Flaxseed)	HC (Wash out)	HD (Placebo)	*p* Value Group 1	OA (Run in)	OB (Flaxseed)	*p* Value Group 2
Endothelial cell biomarkers								
VEGF, pg/mL	57.0 (42.0–3172.0)	41.5 ** (17.0–3063.0)	45.5 (27.0–2765.0)	50.0 (35.0–2791.0)	0.024	50.0 (16.0–103.0)	67.0 (15.0–158.0)	0.168
sICAM-1, ng/mL	141.5 (77.0–262.0)	131.5 (81.0–234.0)	171.0 (40.0–279.0)	231.5 (129.0–276.0)	0.154	148.0 (95.0–295.0)	118.0 (33.0–288.0)	0.118
sVCAM-1, ng/mL	88.5 (54.0–148.0)	91.0 (52.0–201.0)	134.5 (64.0–151.0)	88.5 (35.0–147.0)	0.522	84.0 (35.0–142.0)	74.0 (22.0–121.0)	0.577
oxy-LDL, ng/mL	103.0 (94.0–623.0)	103 (84.0–575.0)	100.5 (86.0–271.0)	104.0 (70.0–212.0	0.795	95.0 (80.0–267.0)	97.0 (79.0–234.0)	0.646

** Significance difference run-in vs. flaxseed.

**Table 3:** Changes in inflammatory and lipid parameters throughout the study. The data are given as medians and range. Abbreviations: hsIL-6, high-sensitivity IL6; hsIL-1beta, high-sensitivity interleukin 1 beta; hsCRP, high-sensitivity CRP; hsTNF alpha, high-sensitivity TNF alpha; HDL, high density lipoprotein cholesterol; LDL, low density lipoprotein cholesterol; TC, total cholesterol; TG, triglycerides. The data were analysed with Friedman test with Dunn post hoc test. Wilcoxon signed-rank test. The data of lipid and inflammatory parameters in the group 1 were presented in manuscript [7]. Data supporting the conclusions of this article are included within the manuscript.

Parameters	Group 2 (*n* = 11)		
	OA (Run in)	OB (Flaxseed)	*p* Value
hsCRP, µg/mL	400.4 (141.5–798.3)	494.3 (252.3–967.3)	0.840
hsTNF alpha, pg/mL	0.958 (0.517–1.538)	0.858 (0.475–1.299)	0.075
hsIL-1β, pg/mL	0.382 (0.339–0.669)	0.405 (0.327–0.722)	0.174
hsIL-6, pg/mL	9.3 (7.6–25.4)	9.3 (7.5–16.0)	0.562
LDL, mg/dL	150.7 (102.4–182.4)	143.2 (99.3–173.6)	0.067
TC, mg/dL	239.0 (178.0–271.0)	217.0 (120.6–250.0)	0.022
HDL-C, mg/dL	52.0 (41.0–65.0)	55.0 (44.0–63.0)	0.210
TG, mg/dL	167.0 (76.0–267.0)	125.0 (77.0–200.0)	0.013

## Data Availability

The data supporting the conclusions of this article are included within the manuscript. The dataset is available from the corresponding author on request.
